# PEGylated Lipid Nanoparticle Formulations: Immunological Safety and Efficiency Perspective

**DOI:** 10.1021/acs.bioconjchem.3c00174

**Published:** 2023-05-10

**Authors:** Rumiana Tenchov, Janet M. Sasso, Qiongqiong Angela Zhou

**Affiliations:** CAS, a division of the American Chemical Society, 2540 Olentangy River Road, Columbus, Ohio 43202, United States

## Abstract

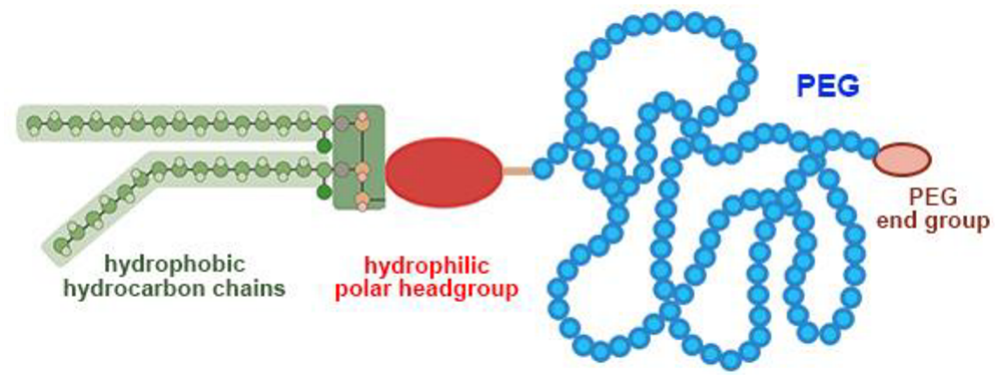

Lipid nanoparticles (LNPs) have been recognized as efficient vehicles to transport a large variety of therapeutics. Currently in the spotlight as important constituents of the COVID-19 mRNA vaccines, LNPs play a significant role in protecting and transporting mRNA to cells. As one of their key constituents, polyethylene glycol (PEG)–lipid conjugates are important in defining LNP physicochemical characteristics and biological activity. PEGylation has proven particularly efficient in conferring longer systemic circulation of LNPs, thus greatly improving their pharmacokinetics and efficiency. Along with revealing the benefits of PEG conjugates, studies have revealed unexpected immune reactions against PEGylated nanocarriers such as accelerated blood clearance (ABC), involving the production of anti-PEG antibodies at initial injection, which initiates accelerated blood clearance upon subsequent injections, as well as a hypersensitivity reaction referred to as complement activation-related pseudoallergy (CARPA). Further, data have been accumulated indicating consistent yet sometimes controversial correlations between various structural parameters of the PEG–lipids, the properties of the PEGylated LNPs, and the magnitude of the observed adverse effects. Detailed knowledge and comprehension of such correlations are of foremost importance in the efforts to diminish and eliminate the undesirable immune reactions and improve the safety and efficiency of the PEGylated medicines. Here, we present an overview based on analysis of data from the CAS Content Collection regarding the PEGylated LNP immunogenicity and overall safety concerns. A comprehensive summary has been compiled outlining how various structural parameters of the PEG–lipids affect the immune responses and activities of the LNPs, with regards to their efficiency in drug delivery. This Review is thus intended to serve as a helpful resource in understanding the current knowledge in the field, in an effort to further solve the remaining challenges and to achieve full potential.

## Introduction

Lipid nanoparticles (LNPs) have been recognized as efficient vehicles to deliver a large variety of therapeutic agents.^1^ Currently in the spotlight as important constituents of the COVID-19 mRNA vaccines, LNPs play a vital role in efficiently protecting and transporting mRNA to cells.^2^ As one of the component lipids on the LNP exterior, PEG–lipid conjugates (PEG–lipids) play a significant role in determining the LNP physicochemical properties and biological relations. The modification of pharmaceuticals with polyethylene glycol (PEG), a flexible, hydrophilic polymer, is a widely implemented strategy to reduce clearance by the reticuloendothelial system, prolong circulation time, improve pharmacokinetics, and enhance drug efficacy.^3−6^

PEGylation (covalently binding PEG to a compound) was initially invented to facilitate protein drugs in avoiding immune response^3,7,8^ but was later discovered to be also quite efficient at enhancing the surface properties of the LNPs by blocking access to their surface through steric obstruction, thus reducing opsonization by blood proteins and clearance by macrophages, yielding longer systemic circulation.^4−6,9^ Because PEGylated lipids prevent aggregation of LNPs, they overall increase their stability. The circulatory half-life of LNPs correlates with various PEG parameters such as the length and concentration of the polymer chains on the LNP exterior, yielding stable LNPs to be prepared by optimizing these parameters.^10^ The enhanced circulation half-life of sterically stabilized LNPs also amplifies their accumulation in cancer tissues by the enhanced permeation and retention (EPR) effect, additionally improving their performance.^11^ PEGylation has progressively become a gold standard for the development of novel drug delivery systems because of the benefits in effectively extending the circulation time, increasing the stability of drug carriers, and greatly improving their pharmacokinetics and efficiency.^12,13^

Along with establishing the benefits of PEG conjugates in extending circulation kinetics and improving pharmacokinetics, it has been reported that unanticipated immune reactions have taken place against PEG-conjugate-bearing nanocarriers. One such unsuspected response is the rapid clearance of PEGylated nanocarriers upon repeated administration, known as the accelerated blood clearance (ABC).^14,15^ ABC comprises production of anti-PEG antibodies at the first injection, which activates accelerated blood clearance upon subsequent injections.^14−17^ Another unanticipated immune response is a hypersensitivity reaction referred to as complement activation-related pseudoallergy (CARPA), which significantly reduces the safety of PEGylated nanocarriers and also correlates with reduced efficacy of PEGylated pharmaceuticals in clinical trials.^18−20^ Such immunogenicity and adverse reactivity effects of PEGylated nanocarriers are a potential concern for the clinical use of PEGylated therapeutics. The growing awareness that anti-PEG antibodies may have a clinical impact resulted in the U.S. Food and Drug Administration (FDA) calling for the measurement of anti-PEG antibody responses in new drugs that contain PEG molecules.^21^

**Figure 1:**
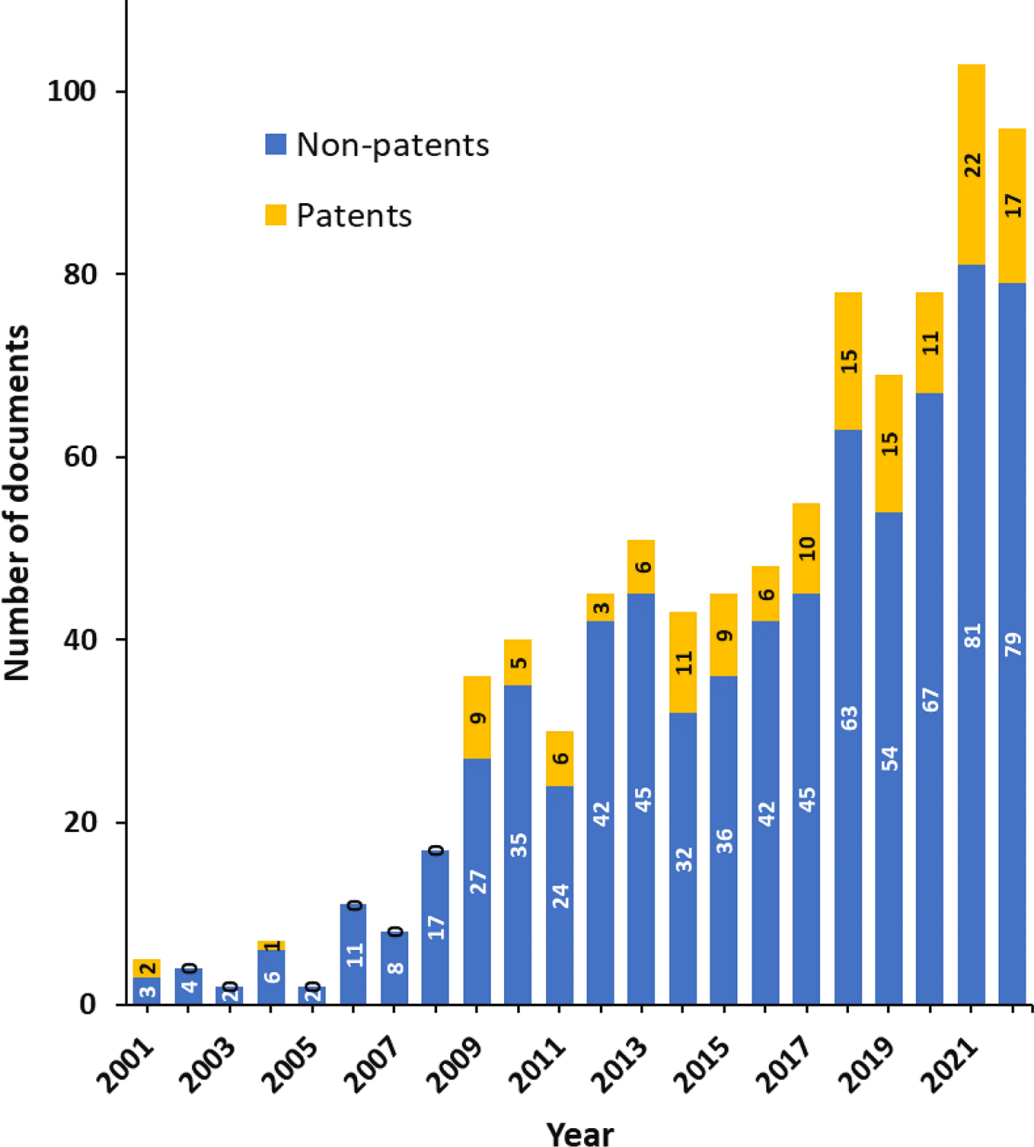
Yearly growth of the number of documents (patents and nonpatents) in the CAS Content Collection related to the PEG–lipids immunologically induced adverse effects such as anti-PEG antibodies generation, ABC, and CARPA.

The initial reports on PEGylation-induced immunogenicity^14,22−24^ sparked considerable interest among research scientists, which was further boosted by the recent use of PEG–lipids in the COVID-19 mRNA vaccines.^1,2^ A search in the CAS Content Collection^25^ identified nearly 900 documents, including ∼150 patents, related to the PEG–lipids immunologically induced adverse effects such as anti-PEG antibodies generation, accelerated blood clearance, and complement activation-related pseudoallergies (Figure 1).

In addition to the above-mentioned immunologically based adverse effects, PEGylation of nanoparticles may lead to other undesirable effects in drug delivery. PEGylation of LNPs has been reported to reduce cellular uptake and endosomal escape in some cases, thus lowering the overall efficiency.^26^ PEG shell provides a steric barrier to efficient binding of particles to the cell and hinders endosomal release by obstructing membrane fusion between the LNPs and the endosomal membrane. That is why the type and amount of PEG–lipids have to be carefully adjusted considering the delicate balance between sufficient stealth and stabilization effects, on the one hand, while not hindering cargo release, on the other. This phenomenon has been referred to as the “PEG Dilemma”.^27^

Several promising yet still unsatisfactory alternatives to PEG have been also examined. To achieve an adequate alternative, further joint research efforts of polymer chemists and drug delivery scientists are necessary to design, synthesize, and evaluate successful alternatives to PEG.^28^

Along with documenting the adverse immune responses triggered by the PEGylated nanocarriers, data have been accumulated for explicit yet sometimes controversial correlations between various structural parameters of the PEG–lipids, the properties of the PEGylated LNPs, and the magnitude of the observed adverse effects.^29−32^ Detailed knowledge and comprehension of such correlations are of foremost importance in the efforts to diminish and eliminate the undesirable immune reactions and enhance the immunosafety and efficiency of the PEGylated medicines.

Here, we present an overview based on analysis of data from the CAS Content Collection regarding the PEGylated LNP immunogenicity and overall safety concerns. A detailed summary has been compiled outlining how various structural parameters of the PEG–lipids affect the immune responses and overall activities of the PEGylated LNPs, with regards to their efficiency in drug delivery. We anticipate this Review would be a helpful resource in comprehending the current knowledge regarding the immunosafety and efficiency of drug delivery systems comprising PEG–lipid conjugates, in an effort to further address the remaining challenges in the field.

## PEG–Lipid Immunogenicity Basis

### Allergic Reactions to the mRNA COVID-19 Vaccines

The recent approval of the two mRNA COVID-19 vaccines, mRNA-1273 and BNT162b2, brought the PEGylated LNP into the spotlight.^2,33^ LNP mRNA vaccines for SARS-CoV-2 include a PEG–lipid conjugate to stabilize the LNPs.^2,34,35^ The included amounts are 50 and 117 μg of the ALC-0159 and PEG2000-DMG PEG lipids per dose of the BNT162b2 (Pfizer-BioNTech) and mRNA-1273 (Moderna) vaccines, respectively.^36,37^

As one of the unsolicited adverse events of the mRNA COVID-19 vaccines, hypersensitivity reactions were reported during the clinical trials.^38,39^ After public vaccination began, cases of anaphylaxis occurred after administering the Pfizer-BioNTech and Moderna COVID-19 vaccines, with the rates of 11 and 2.5 cases of anaphylaxis per million doses, respectively, as of January 2021, with considerable part of the cases having a history of anaphylaxis.^40,41^ As of April 2022, the rate of anaphylaxis has been estimated between 2.5 and 4.7 per million.^42^ 71% of the cases occurred within 15 min of vaccination.^40,41^ Some of the reactions, which have been observed with SARS-CoV-2 mRNA vaccines, have been also been reported for the systemically administered drug Doxil; however, the frequency of these reactions to vaccines has been much lower than that to Doxil.^42^ Generally, the serious allergic reactions upon administration of all COVID-19 vaccines are very infrequent, but slightly higher than the traditional vaccines. The risk of developing such reactions among COVID-19 vaccinees has been reportedly highest in the immediate time period after the vaccination, and it is higher in women as well as in those with a history of allergy.^43^

**Figure 2:**
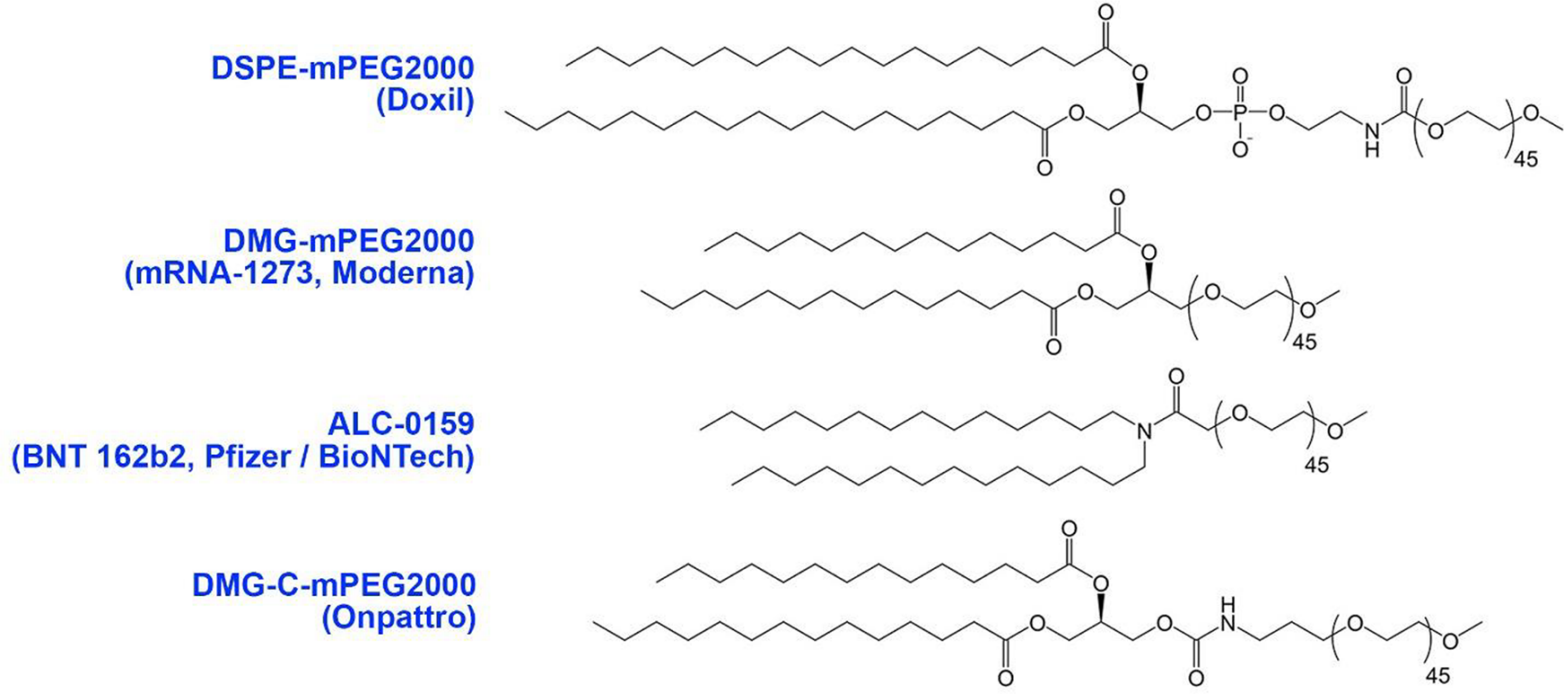
Exemplary PEG–lipid structure used in marketed formulations (DMG-C-mPEG, mPEG-carbamate-1,2-dimyristoyl-*sn*-glycerol).

Studies have reported that mRNA-1273 happen to induce more anti-PEG antibodies than BNT126b2.^44,45^ Because the PEG–lipids in both vaccines use the same PEG-2000 conjugate and the same saturated 14:0 hydrocarbon chains, and the same methoxy terminal groups, the difference in their immunogenicity points to the possible influence of the (i) lipid polar group, (ii) hydrocarbon chains–polar headgroup linkage, or (iii) PEG–lipid linkage interface on the reactivity to mRNA vaccine formulations (Figure 2). Another likely reason could be the higher administered dose of mRNA-1273 (100 μg) as compared to BNT126b2 (30 μg).^38,39^

### PEG–Lipid Immunogenicity

Free PEG is supposed to be nonimmunogenic and nonantigenic, classified under the Generally Recognized As Safe (GRAS) category by the FDA;^46^ hence the antigenic determinant for anti-PEG antibodies has been implied to be located at the linker between PEG and other materials. Thus, the anti-PEG antibody epitope is supposedly the interphase between a hydrophobic core and conjugated PEG groups.^47^ In this respect, PEG supposedly functions as a hapten, that is, a substance that provokes immune response only when conjugated to a carrier. Indeed, studies suggest that PEG-associated immunogenicity is related to attributes of the counterpart of the PEG-conjugates, and not to PEG itself.^30^ For example, while liposomes comprising PEG–DSPE exhibit ABC phenomenon, same mole fraction of the same PEG lipid in polymeric micelles does not exhibit it.^30^ Noteworthy, recent studies consider the early conclusions about the inert nature of PEG misguided and a reason for the delayed scientific efforts to identify and understand anti-PEG antibodies.^48^

### Accelerated Blood Clearance (ABC)

PEG has a long history of safe successful use in humans, being classified under the GRAS category by the FDA. As such, PEG has been used in numerous cosmetic, household, and medical over-the-counter products. PEGylation (covalently binding to PEG) has been considered a breakthrough in pharmaceutics for its capacity to significantly improve pharmacokinetics of drug delivery systems. Despite the frequent use of PEG to enhance pharmacokinetics, rapid clearance of certain PEGylated formulations upon repeated administration has been reported, which correlates with reduced efficacy of these therapeutics in clinical trials. Such anti-PEG immunity is typically robust but short-lived.^14,15^ In animal models, a second dose of PEGylated LNPs, injected in a 5–12 days’ time interval, was cleared fast from the blood circulation. The ABC phenomenon exhibited two distinct phases, induction and effectuation. During the first phase immediately after injection of the initial LNP dose, the biological system is “primed”. The second phase takes place 3–7 days after the initial injection in which a later dose(s) of PEGylated LNPs is rapidly eliminated from systemic circulation.^14,29,49,50^

Such ABC has been associated with the induction of PEG-specific antibodies, IgM, IgG, and IgE.^17,51−55^ The reported anti-PEG antibody response is predominantly IgM; yet the development of anti-PEG IgG and others has also been described.^17,47,50,56−60^ Noteworthy, anti-PEG antibody-mediated complement activation may also be involved in the clearance of repeatedly administered PEGylated therapeutics. Antibodies, particularly IgM, can efficiently activate the complement system and facilitate particle phagocytosis and clearance.^61,62^ The presence of anti-PEG antibodies has been reported to decrease the circulation half-times of PEGylated agents by 2–10-fold on average and to increase the hepatic and splenic accumulation by 2–5- and 1–2-fold, respectively.^50^ In addition to the accelerated removal of PEGylated medicines from blood, various types of anti-PEG antibodies were reported to contribute to the hypersensitivity reactions and premature drug release from PEGylated carriers.^31,51−55^

It has been suggested that the anti-PEG antibody induction follows a type 2 T-cell independent mechanism.^15,63^ The initial dose of a PEGylated drug formulation enters the spleen, comes in contact with marginal zone B cells, and cross-links the surface antibodies, activating the production of PEG-specific IgM antibodies. Further, the induced anti-PEG IgM bind to subsequent doses of PEGylated drug in the circulation and activate complement binding, eventually causing hepatic clearance through Kupffer cell uptake.^15^

### Complement Activation-Related Pseudoallergy (CARPA)

Hypersensitivity reaction, termed complement activation-related pseudoallergy, is another adverse immune reaction to PEGylated liposomes.^64^ This effect is in some sense opposite to the ABC phenomenon because it takes place at the first treatment and the symptoms typically diminish or disappear upon subsequent treatments; hence such an immunological response is called pseudoallergy. Such hypersensitivity reactions to various PEGylated medicines are not characteristic of the classical IgE-mediated allergy.^18^

The CARPA phenomenon has been categorized as non-IgE-mediated pseudoallergy resulting from the activation of the complement system.^20,65−67^ The complement system controls the immune regulation in the body, coordinates the adaptive immunity, and is a key player in the immune defense.^68^

### Pre-existing Antibodies to PEG

Evidence has emerged that anti-PEG antibodies exist among individuals who presumably never received PEGylated drugs.^69,70^ Thus, between 0.2% and 25% of blood donors have been found to exhibit antibodies specific to PEG in their plasma.^71^ A recent study reported that, between healthy individuals, 26.4% have anti-PEG IgM antibodies, 25% have anti-PEG IgG antibodies, and 8.3% have both anti-PEG IgM and IgG antibodies, with the overall percentage of anti-PEG antibodies (IgG or IgM) positive donors thus being 43.1%.^37^

In fact, it needs to be considered that exposure of humans to PEG occurs frequently in daily life, via cosmetics, household products, and common medicines such as laxatives and others, food packaging, etc. Such exposure might be responsible for certain “immunization” that explains, at least partly, the presence of PEG-specific antibodies in the blood of healthy individuals.

## PEG–Lipid Structure, Correlation to Immunogenicity and Efficiency

**Figure 3:**
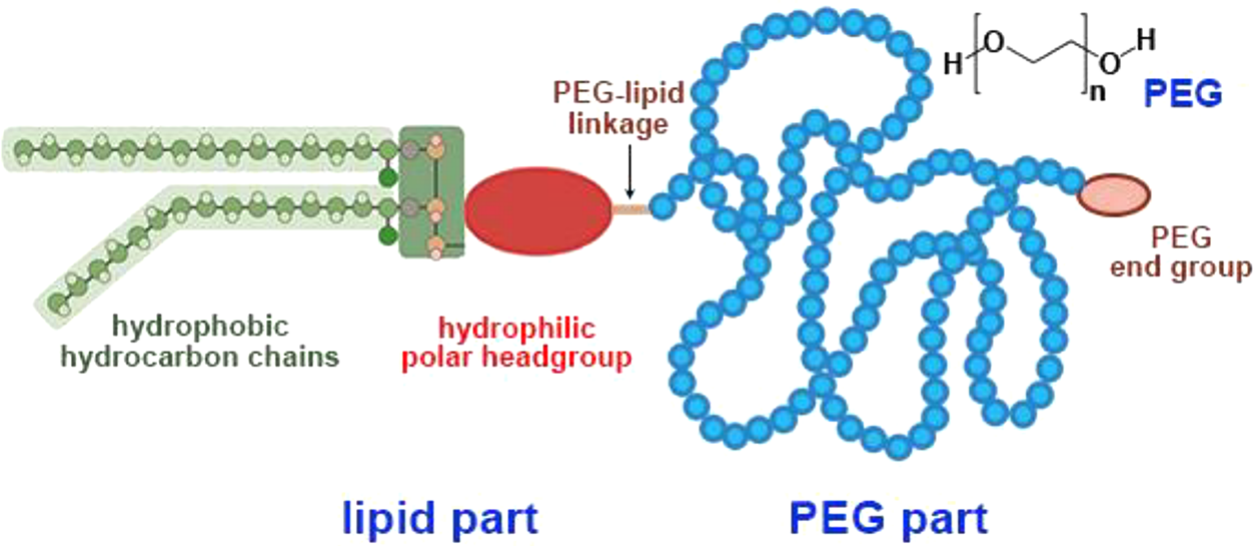
Scheme of the PEG–lipid structure. The chemical structure of the PEG repeats is illustrated in the upper right corner. (In the lipid part, instead of the most widely used dialkyl hydrophobic moiety, in some cases a cholesterol moiety is used; see Figure 4E for an example.)

Various physicochemical parameters of PEGylated LNPs such as particle size,^72−74^ lipid bilayer rigidity and curvature,^75^ PEG density and surface charge,^61,76^ and chemical features of the terminal (end) groups^76−78^ have been reported to affect their immunogenicity, the ABC and the CARPA phenomena.^50^ Specifically, various parameters of the PEG–lipid chemical structure including PEG size and architecture, lipid structure including hydrocarbon chains, headgroup, lipid–PEG linkage, and PEG terminal groups (Figure 3), are significant determinants of the PEGylated LNPs safety and bioactivity.

### Structure of PEG–Lipids

#### PEG Part: Density, Length, Branching, and Terminal Groups

**Figure 4:**
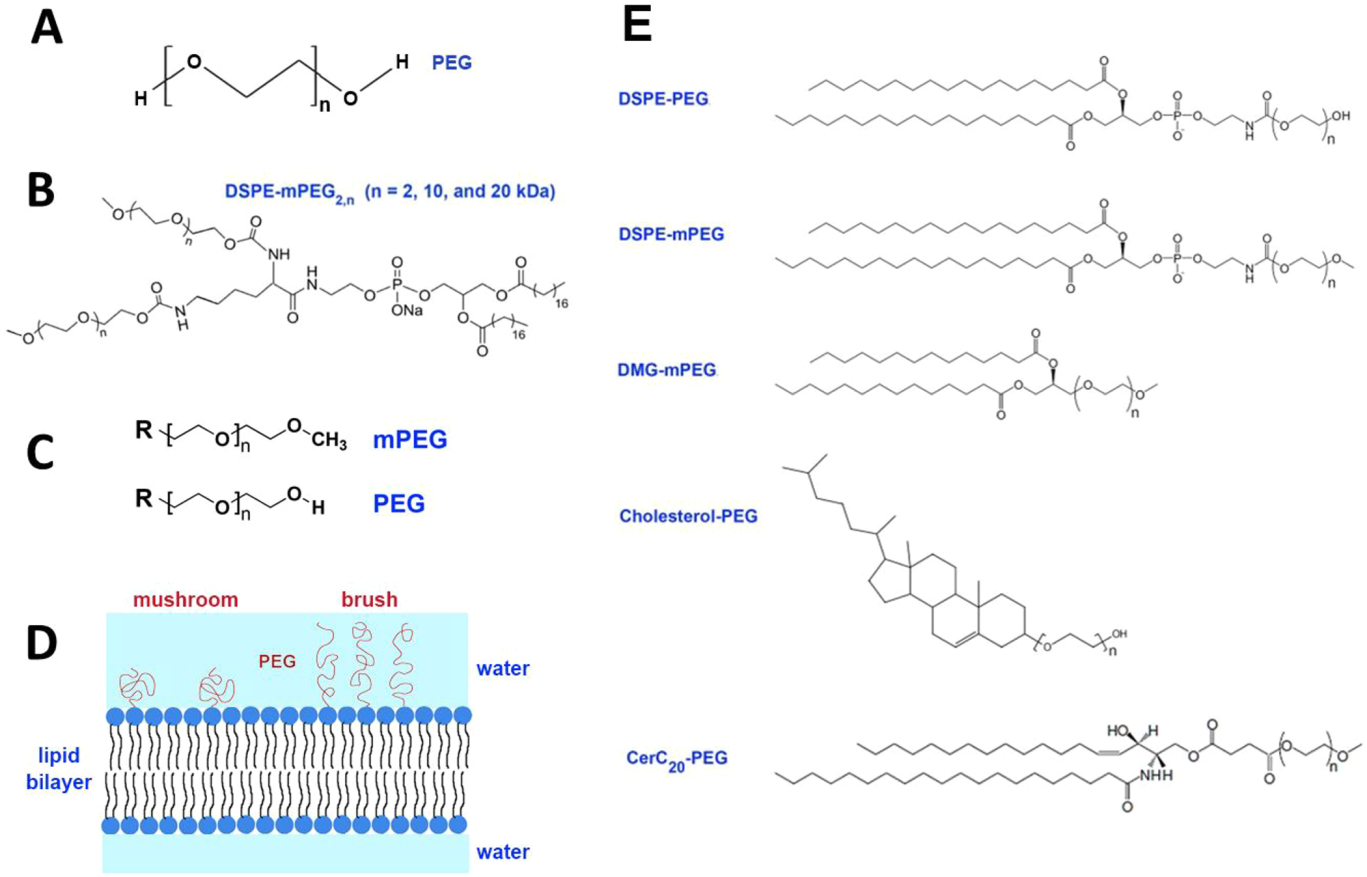
(A) Chemical formula of polyethylene glycol (PEG); (B) chemical structure of an exemplary branched PEG lipid, distearoylphosphatidylethanolamine-mPEG_2,*n*_ (DSPE-mPEG_2,*n*_); (C) PEG and mPEG terminal groups; (D) mushroom versus brush PEG chain configurations depending on PEG density; and (E) exemplary structures of the widely used PEG–lipids in LNP formulations according to the CAS Content Collection.

Poly(ethylene glycol) (PEG) [HO(CH_2_CH_2_O)_*n*_H] (CAS registry number 25322-68-3) (Figure 4A) is a hydrophilic nonionic polymer characterized with a wide range of linear or branched chains of different chain lengths and molecular weights (MWs).^12^ The stealth characteristics of PEG are due to certain molecular characteristics: (i) PEG is extremely hydrophilic, with each ethylene glycol subunit bordered by at least 2–3 water molecules;^79,80^ therefore, PEG coating generates a hydration shield with a large excluded volume that sterically stabilizes LNPs by preventing binding to the underlying core via hydrophobic or electrostatic interactions;^81,82^ and (ii) PEG chains are highly flexible and mobile, with a large number of polymer chain conformations; thus, any reduction in the conformational freedom of PEG caused by intruding macromolecules would be thermodynamically unfavorable.^83^ These aspects strongly restrain interactions between PEGylated surfaces and the biological environment.^50^ Even though PEG is considered of low immunogenicity, or even nonimmunogenic, there is growing evidence that it initiates immunogenic reactions, especially when conjugated to other materials such as proteins and nanocarriers.^84^

##### PEG Length

The biological performance and efficacy of PEGylated nanocarriers are critically dependent on the PEG chain length. A higher PEG length offers enhanced hydrophilicity and flexibility and better steric hindrance, thus avoiding unwanted interactions with biomolecules.^85,86^ Still, there exists a limit for the PEG length above which the PEGylation effect on protein adsorption becomes negligible.^86^ Generally, larger PEGs (20–50 kDa) are used in low molar mass drugs (e.g., oligonucleotides, siRNA, and small molecules), which enlarge the size of the pharmaceutical carrier, thus making it possible to avoid renal clearance. Conversely, smaller PEGs (1–5 kDa) are used in larger drug formulations: antibodies and nanoparticles.^87^ There is growing alertness of systemic allergic reactions to PEGs, sometimes leading to severe anaphylaxis.^88^ The potential of PEG to induce anaphylaxis has been reported to increase with higher MWs and concentration. Thus, risk of allergic sensitization is higher with formulations comprising higher MW PEG such as PEG3350–PEG5000 than with PEG2000.^88,89^ PEGs with molecular weights of 3350 and 4000 Da are responsible for the majority of registered cases of hypersensitivity reaction.^89^ Indeed, PEG5000-modified carrier has been reported to exhibit a lower protein adsorption capacity as compared to PEG2000, which could extend its circulation time,^90^ but also increase its potential to induce ABC phenomenon. In the course of the ABC phenomenon, PEG can affect B cells because of its three attributes: large molecular weight, efficient surface alignment, and long circulation time of the carrier.^91^ Furthermore, laxatives containing PEG3350 have been reported to cause anaphylaxis in children.^92^

It has been reported that PEGs with a lower MW can permeate the skin and mucosa more efficiently than those of a bigger MW, which escalates the risk of sensitization; PEGs with a high MW can trigger hypersensitivity reaction at low concentrations upon sensitization as compared to low MW PEGs.^93^

Another report claimed that the spatial conformation of PEG conjugates with small molecular weight PEGs (PEG400, PEG600, and PEG800) in PEG–cholesteryl carbonate (PEG–CHMC) seem to be more likely to induce a strong ABC phenomenon.^91^

Thus, the effect of the PEG length on the ABC phenomenon seems to be biphasic; both long-chain and short-chain PEG conjugates are more likely to induce a strong ABC phenomenon as compared to the medium-chain PEGs. Generally, studies have shown that LNPs comprising PEG2000–lipid conjugates are the optimal choice for an adequate compromise between the antiopsonization approach, effective targeting, and diminishing ABC effect.^94^

##### PEG Architecture: Linear versus Branched

PEG architecture is another noteworthy determinant of the performance and efficacy of the PEGylated nanocarriers. Branched DSPE-mPEG_2,*n*_ (*n* = 2, 10, and 20 kDa) (Figure 4B) nanocarriers exhibited physical and chemical properties comparable to those of the nanocarriers comprising the linear DSPE–mPEG2000 with respect to their size, polydispersity, and zeta potential, but did not provoke the ABC after recurrent injections.^95^ A possible reason is that the carriers generated markedly lower levels of anti-PEG IgM than linear PEG nanocarriers and did not actuate the complement system. Furthermore, the branched DSPE–mPEG_2,*n*_-modifed liposomal doxorubicin exhibited better in vivo antitumor effects than did linear DSPE–mPEG2000-modifed liposomes.^95^

The branched (mPEG114)_2_–DSPE lipid conjugate has been reported to confer the highest stealth properties to LNPs (∼31-fold lower cell association as compared to the non-PEGylated LNPs) with respect to all PEGylating agents tested including various lipid hydrophobic moieties (DSPE vs cholesterol vs cholane), mPEG of different MWs (2 kDa vs 5 kDa), and linear versus branched PEG moieties.^96^ However, when optimizing PEG–lipid performance, its structural parameters need to be considered in conjunction, because, for example, pharmacokinetic experiments demonstrated that compounds having cholesterol as their lipid part produce PEGylated LNPs with a longer residence time in the circulation and higher bioavailability among the formulations described above. LNPs comprising mPEG114–Chol exhibited 3.2- and ∼2.1-fold higher area under curve (AUC) than did non-PEGylated LNPs and branched (mPEG114)_2_–DSPE-coated LNPs, respectively, due to the high stability of this coating agent. By assessing the PEGylating agents comprising the same size linear 5 kDa PEG derivatives, linear mPEG114–DSPE produced LNPs with the best in vitro stealth performance. Nevertheless, the in vivo AUC of LNPs comprising linear mPEG114–DSPE was lower than that reported for LNPs comprising linear mPEG114–Chol.^96^

##### PEG Terminal Group

Functional terminal groups appended to PEG chains are another factor that affects their immunogenicity. Methoxy-PEG (mPEG) is a PEG chain having one terminal functional group blocked with a methyl group (Figure 4C). The terminal group of PEG has been found to modify PEG immunogenicity, with hydroxy-PEG reportedly less immunogenic than methoxy-PEG.^77^ Antibodies with a high affinity for methoxy groups were suggested to be responsible for the immunogenicity and loss of efficacy of mPEG conjugates. Using functionally activated HO-PEG instead of mPEG in conjugates intended for clinical use might reduce this adverse effect. PEGs with butoxy terminal groups are reported to be more immunogenic than methoxy-PEGs.^31,77,97^

In another study, PEG–lipids with different terminal functional groups, including methoxy (OCH_3_), amino (NH_2_), carboxyl (COOH), and hydroxyl (OH) moieties, were compared with respect to their anti-PEG IgM production and clearance.^98^ The respective LNPs were of similar size, ∼100 nm. All PEG–lipids induced anti-PEG IgM production, with the hydroxy-PEG being the least immunogenic and least antigenic, yet undergoing more rapid clearance as compared to the methoxy-PEG. This is a likely result of the tendency of the hydroxyl groups to activate the complement system, a major mechanism for the clearance of foreign materials.

##### PEG Density

PEG surface density, along with PEG length, is another critical parameter modulating the biological performance and efficacy of PEGylated nanocarriers, and the resultant conformations adopted by the conjugated PEG chains control their biochemical and physicochemical characteristics, including protein adsorption and aggregation level. It has been reported that grafted PEG can arrange into two different conformations, “mushroom” and “brush” (Figure 4D), correlated with the PEG surface density. At low surface density, PEG is in a “mushroom” configuration, in which the polymer chains are separated and arranged close to the surface, while at higher density, the polymer chains are closer and arranged into a straight (brush-like) conformation, thus producing a larger hydrophilic barrier leading to a lower protein adsorption.^99^ PEG conformation can be characterized by Flory radius, which identifies the minimum distance between grafted polymer chains required for allowing a mushroom conformation.^100−103^ Different topologies of the polymer configuration have been reported depending on the correlation between the grafting density and the Flory radius. These topologies significantly affect the pharmacological parameters of the PEGylated drug carriers, such as uptake, stability, and pharmacokinetics. Noteworthy, PEGylation needs to be carefully optimized from a drug targeting viewpoint.^104^ Variations of the PEG surface densities could impact the circulation time of PEGylated LNPs after the initial injection, thus eliciting control over PEG-associated ABC.^105^

Table 1 summarizes the reported effects of the PEG part structural parameters on the immunological adverse effects and activity of the PEGylated LNPs, as discussed above.

**Table 1:** Effect of the PEG–Lipid Structural Parameters on Their Immune-Mediated Adverse Effects and Activity – PEG Part

structural parameters/factors	immune-mediated adverse effects/safety/activity aspects
PEG Part	
PEG length	•formulations comprising higher MW PEG (3350–5000) represent a higher risk of allergic sensitization than PEG2000^88^
	•most of the cases of hypersensitivity reaction are related to PEGs with MWs of 3350 and 4000 Da^89^
	•PEG–cholesteryl carbonate (PEG–CHMC) with small MWs (PEG400, PEG600, and PEG800) induce a strong ABC phenomenon^91^
	•PEG5000 has a lower protein adsorption capacity than that of the PEG2000-modified carrier^90^
	•PEG2000–lipid conjugate is the best choice for an adequate trade-off between avoiding the opsonization strategy and active targeting^94^
PEG architecture: linear versus branched	•branched DSPE–mPEG_2,*n*_ produced a lower amount of anti-PEG IgM than did the linear version; it does not trigger ABC and does not activate the complement system^95^
	•DSPE–mPEG_2,*n*_-modifed liposomal doxorubicin is a more efficient antitumor formulation than liposomes comprising the linear DSPE–mPEG2000^95^
	•LNPs comprising the branched (mPEG114)_2_–DSPE have better stealth properties^96^
PEG terminal group	•hydroxy-PEG is less immunogenic than methoxy-PEG,^77,97^ but undergoes more rapid clearance^98^
	•butoxy-PEG exhibits higher immunogenicity than the methoxy version^31,77,97^
	•with respect to anti-PEG IgM production leading to ABC, the PEG terminal groups arrange: OH < NH_2_ < COOH < OCH_3_^98^
PEG density (percentage in LNPs)	•PEG density exhibits a biphasic effect with respect to immunogenicity: 5 mol % mPEG2000–DSPE provokes maximum ABC, while at lower or higher surface densities the ABC is lower^106^

#### Lipid Part: Hydrophobic Hydrocarbon Chains, Polar Headgroup, Chain–Headgroup Linkage

##### Lipid Chain Length

The lipid hydrophobic chain structure in PEG–lipids affects the LNPs biological activity. An aspect of the lipid chain length effect on the PEGylated LNP performance is via the PEG–lipid “sheddability”, that is, its ability to leave the lipid bilayer upon circulation.^107,108^ Because the PEG–lipid is anchored into the lipid bilayer via its hydrophobic chains, PEG–lipids with longer chains are more tightly bound and thus less likely to dissociate from the LNP, while shorter-chain PEG–lipids are expected to more easily dissociate from the bilayer^109^ and can thus attenuate the occurrence of the ABC phenomenon. Indeed, the desorption rate has been reported to well correlate with the lipid chain length.^110−112^ The PEG–lipid desorption from LNPs in circulation was reported to be 45% for PEG–lipids bearing C14 dialkyl chains and only 1.3% and 0.2% for PEG–lipids bearing C16 and C18 dialkyl chains, respectively, as measured 1 h after administration.^111^ The short-chain PEG–lipids are prone to dissociate into the endogenous lipid-associating bodies such as lipoproteins, extracellular vesicles, and blood proteins such as albumin comprising hydrophobic pockets.^113^ The ABC phenomenon was indeed avoided by the gradual dissociation of PEG from the particles, when using shorter chain C14 PEG–lipids.^23,60^

Variation of the acyl chain length of the PEG–lipids modulates the stability of the incorporation of these lipids in the particles, which leads to a modification of the pharmacokinetics. Application of a PEG–lipid comprising short (C14) acyl chains dissociating from LNPs in vivo with a halftime of <30 min results in optimum bioactivity in certain cases such as hepatocyte gene-silencing.^114^ The generation of anti-PEG antibodies in mice has been found to be affected by the rate at which PEGylated lipid has been shed from LNPs, with fast shedding resulting in a lower amount of anti-PEG antibodies.^110^

Stealth PEGylated liposomal formulations of doxorubicin or irinotecan carriers incorporate a long-chain 1,2-distearoyl-*sn*-glycero-3-phosphoethanolamine–poly(ethylene glycol 2000) (DSPE–PEG2000) that is firmly affixed in the lipid bilayer.^4,115^ Conversely, PEG lipids with shorter tails stay in the LNPs upon production but are fast released in vivo.^116,117^ Thus, DSPE–PEG2000 remains in LNPs with a half-life of ∼25 h, whereas a neutral-lipid 14-carbon chain PEG molecule (DMG–PEG2000) is fast released with a half-life of ∼1.3 h,^118^ permitting more efficient interaction of the LNPs with target cells.^108,119^

Generally, because LNPs interact with their environment via their hydrophilic exterior surface, most of the attempts in enhancing the lipids’ efficiency in drug delivery systems have been aimed at the synthesis of amphiphiles with different varieties of polar groups. Generally, less attention has been paid to the role of the lipid hydrophobic region, that is, the lipid chain length, saturation, and branching. In fact, recent studies suggest that the nonpolar parts modulate in a significant way the bioactivity of the lipidic nanocarriers by modifying important physicochemical properties such as membrane fluidity and curvature as well as phase propensities, thus regulating fusogenicity.^120,121^ Evidence has been accumulated for a relationship between bioactivity and lipid phase behavior, which, as is well-recognized, is strongly modified by the hydrophobic parts of the lipid molecules.^122,123^ For example, for certain cationic lipids, it has been reported that their activity as genetic vectors rises upon increasing chain unsaturation and decreases at higher chain length, with maximum transfection being reported for monounsaturated 14:1 chains.^124,125^

The physicochemical and pharmacological characteristics of LNPs comprising a set of monomethoxy-poly(ethylene glycol)–lipids (mPEG–lipids) showed that branched mPEG lipid correlates with best LNP stealth properties in the in vitro experiments. The pharmacokinetic experiments demonstrated that a cholesterol anchoring group is favorable with respect to the residence time in the circulation and the systemic bioavailability.^96^

##### Lipid Headgroup and Charge

It has been reported that the phosphate group net anionic charge of the mPEG conjugate played a significant role in activation of complement and anaphylatoxin generation. Thus, methylation of the phosphate oxygen and the resultant elimination of the negative charge was demonstrated to totally prevent complement activation.^126^ It has been thus hypothesized that complement activating natural anti-phospholipid antibodies (IgG and IgM) may need both the phosphocholine headgroup of the dipalmitoylphosphatidylcholine (DPPC) and the anionic moiety of phospholipid–mPEG in a certain spatial relationship orienting the antibody into a complement activating posture to enable binding to PEGylated liposomes.^126^ Such knowledge would provide a rational basis for formulating safer drug delivery vehicles by avoiding complement activation.

PEGylated carriers prepared with negatively charged phospholipids have been reported to exhibit a stronger vasoactive outcome than those comprising neutral phospholipids, implying that charged vesicles exhibit a stronger immunostimulating effect via the complement activation than the neutral vesicles.^127^

##### Hydrocarbon Chains–Polar Headgroup Backbone Linkage

Changing the ester linkage into a carbamate linkage resulted in the formation of unstable vesicles, demonstrating that the lipid linkage is a significant parameter in lipid architecture.^128^

##### Lipid–PEG Linkage

The lipid–PEG linkage could also modify the PEG–lipid performance. PEG–lipid derivatives in which a single ester bond connects the PEG and lipid parts so that they could be easily de-PEGylated by esterase cleavage of PEG in circulating blood have been shown to significantly attenuate the occurrence of the ABC phenomenon.^129,130^ Another cleavable PEG–lipid derivative exploited a PEG–cholesterol conjugate exhibiting two ester bonds and one pH-sensitive bond, with which no ABC phenomenon was observed upon repeated LNP administration.^131^ Indeed, a degradable linker would possibly result in faster and supposedly more complete shedding.^108^

Other options of lipid–PEG linkages have been also examined. A disulfide-linked DSPE–PEG conjugate has been synthesized and reported to exhibit high sheddability (50% liposome cargo release within ∼1 h).^132^ To enhance the reductive shedding, another type of reduction-sensitive dithiobenzyl carbamate linker was designed to couple PEG to DSPE.^133^ Other sheddable lipid–PEG linkages have been also considered and tested:^108^ dithioester,^134−137^ orthoester,^138^ vinylether,^139−142^ phosphoramidate,^143^ hydrazone,^144,145^ β-thiopropionate,^146,147^ and disulfide.^132,133,148−151^

Table 2 summarizes the reported effects of the lipid part structural parameters on the immunological adverse effects and activity of the PEGylated LNPs, as discussed above.

**Table 2:** Effect of PEG–Lipid Structural Parameters on Their Immune-Mediated Adverse Effects and Activity – Lipid Part

structural parameters/factors	immune-mediated adverse effects/safety/activity aspects
Lipid Part	
lipid hydrocarbon chains: saturated versus unsaturated	•ABC of PEGylated LNPs comprising unsaturated phospholipids is higher than that of saturated phospholipids^75^
lipid anchor: PE^a^ versus diglyceride versus sterol	•LNPs comprising cholesterol–PEG conjugates have a longer residence time in circulation and a higher systemic bioavailability^96^
lipid hydrocarbon chain length	•shorter chains (14C) are better than longer (18C) with respect to safety and efficiency^108,118,119^
	•rapid-shedding PEG–lipid (shorter hydrocarbon chains, DMG–PEG) produce a lower amount of anti-PEG IgM than do slower-shedding PEG–lipid (longer hydrocarbon chains, DSG–PEG)^110^
	•for liposomes comprising PEG–ceramide conjugates with C8, C14, C20, and C24 acyl chains, the shorter chain conjugates undergo rapid release from the LNPs after iv administration, while the longer chain conjugates exhibit stronger anchoring in the lipid bilayers^109^
	•replacing slow-shedding long-chain PEG–lipids (PEG–CerC_20_) with rapid-shedding short-chain PEG–lipids (PEG–CerC_14_) abolishes the immune response to PEGylated liposomes^23^
lipid headgroup charge	•the anionic charge at the phosphate group of phospholipid conjugates plays a significant role in the complement activation and anaphylatoxin production; elimination of the negative charge by methylation prevents complement activation^126^
	•negatively charged PEG lipid with C18-hydrocarbon chains stably associates in lipid particles, while neutral C14 PEG lipid spontaneously shreds out from LNPs^37^
	•neutral PEGylated liposomes result in accelerated clearance as compared to charged cationic anionic liposomes^152^
hydrocarbon chains–polar headgroup linkage	•lipid linkage is important for LNP performance: if the ester linkage is replaced by a carbamate one, unstable vesicles are formed^128^
lipid–PEG linkage	•cleavable PEG–lipid ester linkages significantly attenuate or eliminate the occurrence of the ABC phenomenon^129−131^

a Abbreviations: PE, phosphatidylethanolamine; DSPE, distearoyl PE; PC, phosphatidylcholine; DSPC, distearoyl PC; DOPC, dioleoyl PC; and SOPC, stearoyl-oleoyl PC.

## LNP Composition and Properties

### PEGylated Lipid Molar Part

The effect of the PEG–lipid molar part in the LNPs (i.e., the PEG surface density) on the ABC effect has been reported to be biphasic. Thus, PEGylated LNPs comprising 5 mol % mPEG2000–DSPE provoke a maximum ABC effect in rats; lower or higher PEG surface densities resulted in a lower ABC phenomenon.^153^ Supposedly, a low PEG density has been insufficient for the splenic B cells activation, while a high PEG density caused a reduction of the splenic B cells reactivity.^153^

The presence of PEG–lipid conjugates in the lipid bilayer has been reported to modulate the lipid phase behavior. Thus, for lipids with propensity to nonlamellar phase formation such as phosphatidylethanolamines, low amounts of PEGylated lipids suppress that propensity shifting up the lamellar-inverted hexagonal phase transition temperature. Additionally, short PEG conjugates such as DMPE–PEG550 induce the formation cubic phase at temperatures between the lamellar and inverted hexagonal phases.^154,155^

PEG–lipids participate in LNP self-assembly via the hydrophilic steric barrier formed by PEG chains at the LNP surface.^6,156^ The hydrophilic PEG barrier also supports particle stability by preventing aggregation. The PEG is long-lasting, up to 3 weeks at 4 °C in buffer, exhibiting steady size, polydispersity, zeta-potential, and encapsulation upon storage.^110,113^

### Lipid Composition

The effect of phospholipid composition with specific attention on hydrocarbon chain saturation has been examined in PEGylated LNPs prepared by various phospholipid types including several phosphatidylcholines and a egg sphingomyelin, in rats.^75^ Unsaturated phospholipids triggered a stronger ABC phenomenon than did the saturated ones.

Helper lipids are regularly used in LNP formulations. They are believed to improve stability upon storage and circulation and generally enhance the LNP properties. The helper lipids mostly include sterols, phospho-, and glycolipids.^26^ Cholesterol is one of the most common helper lipids used in LNP formulations. It has been recognized as a powerful regulator of membrane fluidity. In highly fluid membranes, it triggers the formation of a liquid-ordered phase with lower fluidity and lower bilayer thickness. In membranes of low fluidity, it enhances membrane fluidity and the bilayer is thinner.^113,157^ Studies on various membrane compositions with respect to their complement activation abilities suggest that there may be optimal fluidity requirements,^158^ which might be modulated by the cholesterol content. Further, studies show that, while the cholesterol content of ≤45 mol % is not critical for pulmonary vasoactivity, iv injection of lipid carrier with high cholesterol content (71%) can trigger complement activation and anaphylactic shock.^159^ It has been reported that at high cholesterol content the hypertensive lung effect is proportional to the cholesterol content in the liposomal preparations.^160^

LNP size needs to be controlled upon preparation because it is a key determinant in nanoparticle pharmacokinetics, biodistribution, and delivery efficiency.^161,162^ Indeed, the higher size of an antigen means higher surface area available for antibody-specific antigen interaction. Increased vesicle size and polydispersity in large multilamellar liposomes have been reported to enhance the pulmonary hypertensive response, while small unilamellar, monodisperse liposomes had no significant vasoactivity.^160^ Increasing the molar ratio of the PEG–lipid has been correlated with significantly smaller LNPs.^163,164^ In general, the least reactogenic and thus preferred liposomal formulations have been demonstrated to consist of homogeneous, ∼100 nm small unilamellar vesicles with slightly negative zeta potentials.^165^

Further, it has been demonstrated that polymeric micelles with size <31.5 nm can effectively avoid immune cells, while those with size >50.2 nm triggered anti-PEG ABC.^73^ Alternatively, in research using PEGylated liposomes, it has been documented that the size (100, 400, or 800 nm) and charge (+13.15, −46.15, and −1.51 mV) of the initial PEGylated liposome dose did not affect ABC induction,^15^ thus leaving the question of the impact of the properties of the PEGylated particles on the anti-PEG immune response open.^15^

Table 3 summarizes the reported effects of the lipid composition and properties on the immunological adverse effects and activity of the PEGylated LNPs, as discussed above.

**Table 3:** Effect of the Lipid Composition and Properties on the PEGylated LNPs Immune-Mediated Adverse Effects and Activity

structural parameters/factors	immune-mediated adverse effects/safety/activity aspects
LNP Composition and Parameters	
lipid composition	•cholesterol as a helper lipid suppresses protein binding and improves circulation time; it is also essential to particle stability^113,166^
	•high cholesterol percentage in the bilayer membrane (≥70%) causes higher complement activation^167^
	•phosphatidylcholines such as DSPC as helper lipids improve endocytosis of LNPs; substituting DSPC with the unsaturated DOPC and SOPC causes enhanced particle uptake and intracellular delivery^113,164,168^
size and surface charge	•a rise in the PEG–lipid molar part from 1% to 5% correlates with significantly smaller LNPs^163,164^
	•increased vesicle size and polydispersity of large multilamellar liposomes enhance pulmonary hypertensive response, while small unilamellar, monodisperse liposomes had no significant vasoactivity^160^
	•anionic PEG–lipids undergo a drop in particle size with increasing PEG size and molar ratios in the LNPs^169^
	•PEGylated carriers comprising negatively charged phospholipids stimulate complement activation stronger than neutral vesicles^127^
	•the effect of particle size on LNP immunogenicity is controversial and requires further investigations^15^

### Dosage, Regimen, and Type of Administration

The ABC phenomenon and the lipid dosage have been reported to exhibit a strong negative relationship with respect to the initial PEGylated LNP dose.^153,170^ Thus, rats injected with doses >5 μmol lipid/kg did not show elevated anti-PEG IgM. In contrast, at lipid doses <1 μmol lipid/kg, the ABC phenomenon was notably increased, supposedly resulting from the different liposome pharmacokinetics and/or B cells reactivity.^15,29,75^

The incitement and the extent of the ABC have been shown to be related to the time scale between injections. Changes in the encapsulated drug clearance were not observed with PEGylated LNPs repeated injections with time between the first and subsequent injection of less than 2 days or more than 4 weeks.^171,172^ The magnitude of the ABC was the highest when the interval between the two injections was between 4 and 7 days.^14,22,57,153^ The possible reason is that production of anti-PEG IgM was taking place by 3–4 days after the initial dose and the IgM vanished within a half-life of 3 weeks.^17,29,170,173^

The adverse effects caused by PEGylated LNPs have been found affected by the route of administration as well. Bolus intravenous administration has been documented as less causative of ABC relative to the slow infusion, supposedly due to the slower rate of exposure allowing for the elaboration of a strong immune response.^15^ Subcutaneous injection has been reported to increase the ABC of PEGylated nanocarriers upon repetitive administration.^15^ It has been also reported that slow infusion of PEGylated liposomes in pigs suppresses the pulmonary hypertension induced by complement activation, as compared to the bolus intravenous injections.^160^ Elevation of blood anaphylatoxins levels has been suggested as a possible reason for the effect.^174^ Anaphylatoxins levels in the blood are expected to be related to the degree of complement activation provoked by the injected liposomes, as well as the speed of injection. Indeed, clinical protocol for the administration of Doxil, an antitumor doxorubicin formulation comprising PEGylated liposomes, recommends a slow rate of infusion, which possibly accounts to a certain extent for the low or absent infusion reactions in the majority of patients.^29,175,176^

Table 4 summarizes the reported effects of the pharmaceutical parameters on the immunological adverse effects and activity of the PEGylated LNPs, as discussed above.

**Table 4:** Effect of the Pharmaceutical Parameters on the PEGylated LNPs Immune-Mediated Adverse Effects and Activity

structural parameters/factors	immune-mediated adverse effects/safety/activity aspects
Pharmaceutical Parameters	
dosage	•there is a strong negative relationship between the ABC and the lipid amount of the initial LNP dose, regardless of the specific lipid composition; a higher lipid dose correlates with a lower ABC^153,170^
	•the third dose leads to a gradual recovery of the pharmacokinetics (28)
	•phospholipid dose of PEGylated LNPs of <1 μmol lipid/kg induced ABC; higher doses of ≥5 μmol lipid/kg abolished the ABC^15,75^
frequency regimen	•the ABC is at a maximum if the interval between the initial and successive injections is between 4 and 7 days^14,22,57,153^
route and mode of administration	•bolus iv administration is less of a contributor of ABC as compared to slow infusion, supposedly allowing for elaboration of a strong immune response^15^
	•s.c. injections enhances the ABC of PEGylated LNPs upon repetitive administration^15^
	•slow infusion suppresses the pulmonary hypertension induced by complement activation, as compared to the bolus intravenous injections^29,160,177^
	•CARPA can be reduced by premedication with corticosteroids, lowering the infusion rate, or diluting the drug dose^175,176,178^

### Effect of Encapsulated Drug

PEGylated empty LNPs have been reported to induce anti-PEG IgM antibodies production.^57,179^ Conversely, cytotoxic drugs encapsulated in PEGylated LNPs do not produce anti-PEG antibody reactions. Thus, liposomal doxorubicin avoids intense production of anti-PEG IgM and prolongs the half-life of a second dose because B cells with a PEG-recognizing receptor are eliminated by the cytotoxic doxorubicin.^49,61,73,76,128,180−182^ Administration of therapeutic doses of PEGylated LNPs carrying the anti-cancer drugs mitoxantrone or oxaliplatin also do not induce anti-PEG IgM antibody reactions.^170,180,183^

## Landscape View of PEG–Lipids Used in LNP Formulations as Found in the CAS Content Collection

PEGylated LNPs are extensively used in pharmaceutical formulations. The major classes of PEG–lipids used in LNP formulations as represented in the CAS Content Collection are listed in Table 5. An extended version of this table showing also the chemical structures of the PEGylated lipids is included as Table S1. Representative chemical structures of the most frequently used PEG–lipids are summarized in Figure 4E.

**Table 5:**
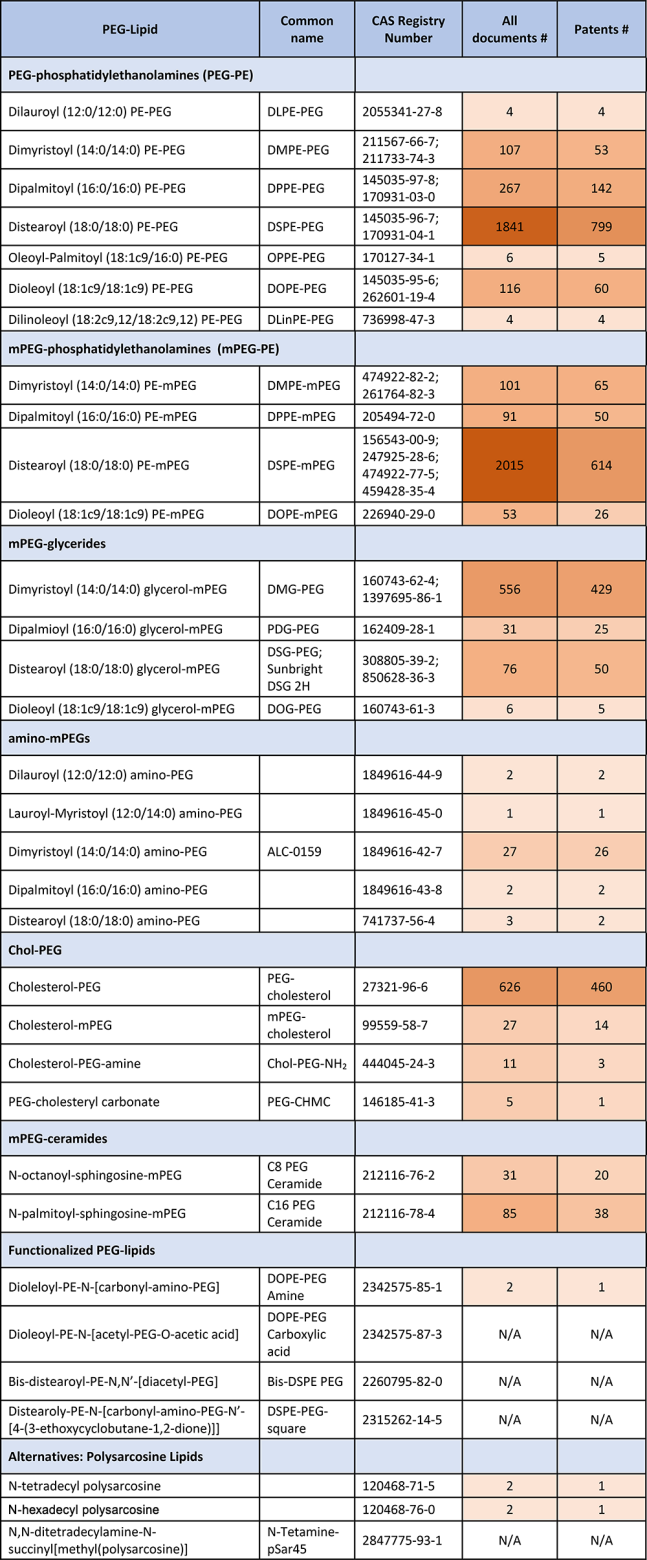
PEG–Lipid Representatives Used in LNPs as Found in the CAS Content Collection with the Respective Number of Documents

**Figure 5:**
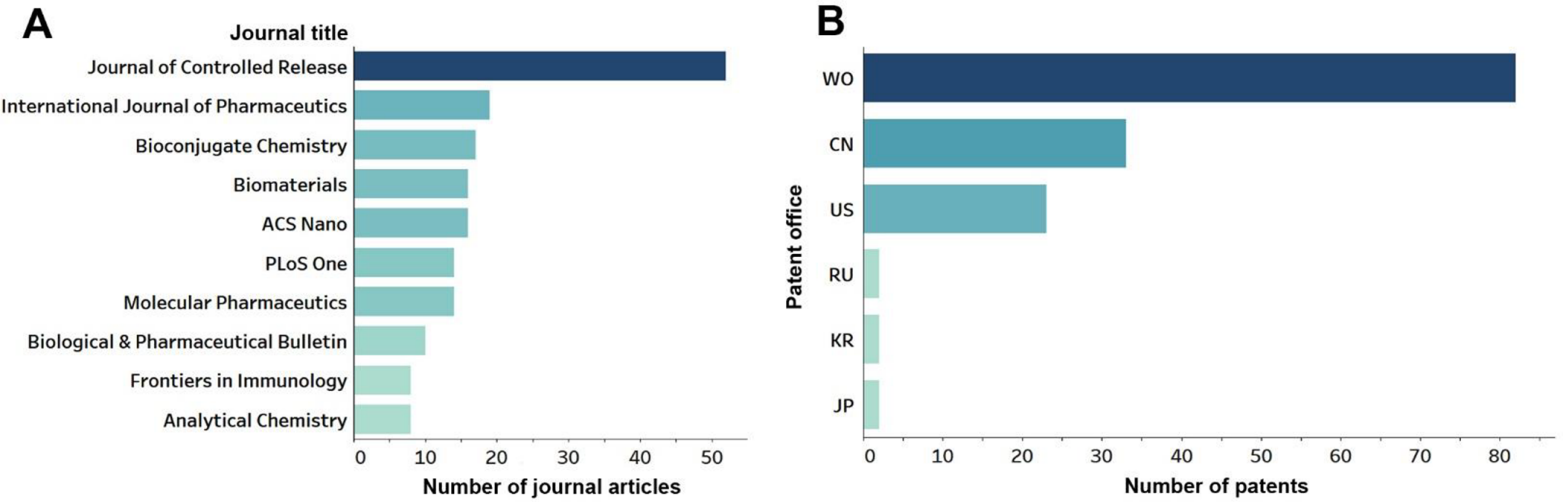
Documents related to the PEG–lipids immunologically induced adverse effects such as anti-PEG antibodies generation, ABC, and CARPA: (A) Top journals publishing articles related to the immunology-related adverse effects; and (B) number of patent applications filed at the top patent offices (the abbreviations on the left indicate the patent offices of World Intellectual Property Organization (WO), China (CN), United States (US), Russian Federation (RU), Korea (KR), and Japan (JP)).

In Figure 5 are depicted the top journals and patent offices responsible for a major part of the documents related to PEG–lipids immunologically induced adverse effects as reflected in the CAS Content Collection. The World Intellectual Property Organization (WIPO) received the highest number of patent applications, followed by the China and U.S. patent offices.

**Figure 6:**
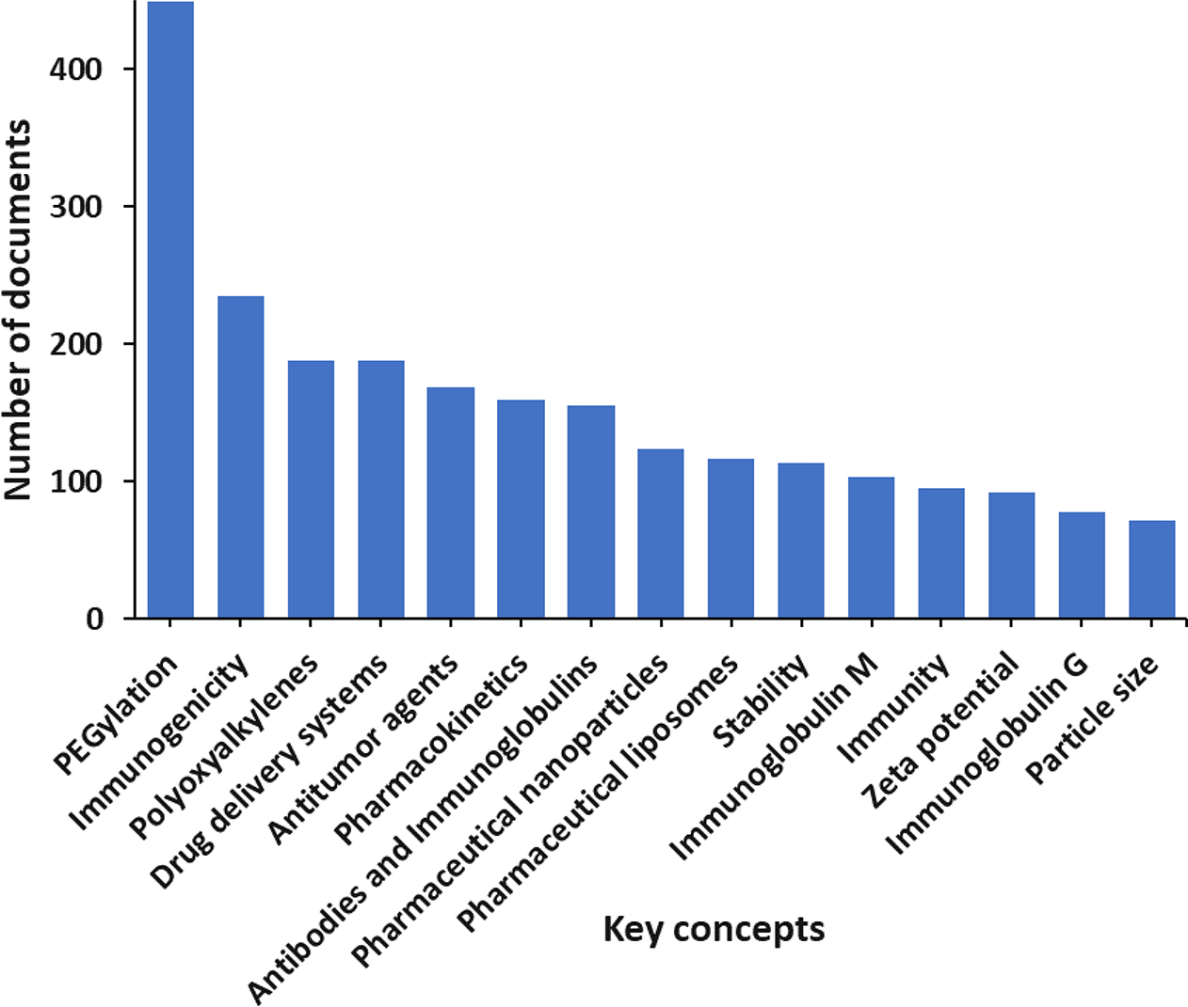
Key concepts related to the PEG–lipids immunologically induced adverse effects such as anti-PEG antibodies generation, ABC, and CARPA according to the CAS Content Collection.

In Figure 6 are depicted the major concepts related to the PEG–lipids immunologically induced adverse effects such as anti-PEG antibodies generation, ABC, and CARPA according to the CAS Content Collection, along with the number of documents that have been discussed. These key concepts reveal the breadth of the field and which areas are receiving the greatest research effort. While key concepts such as PEGylation, polyxoyalkyenes, and drug delivery systems are innate to discover in this search, others such as antitumor agents, immunoglobulin M, and immunoglobulin G reveal more insight into the ongoing research. The key concept antitumor agents show cancer as the largest disease class targeted by PEG–lipid pharmaceuticals. In addition, concepts immunoglobulin M and G reveal the two most common PEG-specific antibodies currently researched for PEG–lipid immunogenicity.

## Alternative Polymers

The reported immunologically based adverse effects associated with the PEG conjugates highlighted the need of searching for alternative polymers instead of PEG. Currently, several hopeful yet imperfect substitutes for PEG have been examined.^28^

The application of other stealth polymers such as poly(oxazoline),^184^ poly(vinyl alcohol),^185,186^ poly(glycerol),^187−191^ poly-*N*-vinylpyrrolidone,^192,193^ poly[*N*-(2-hydroxypropyl)methacrylamide],^194,195^ poly(*N*,*N*-dimethyl acrylamide),^195^ poly(*N*-acryloyl morpholine),^195^ poly(amino acids),^196−198^ and others has been considered and tested as safer nanocarriers.^28,87,191,199−202^ Polysarcosine has been considered in recent patents.^203,204^ Some of these polymers exhibit certain important advantages. For example, poly(oxazoline) demonstrates tunable properties, good biocompatibility, enhanced renal clearance, and favorable biodegradability.^205^ Poly(*N*-vinylpyrrolidone) exhibits lower degradation than PEG under UV or ultrasound irradiation.^87^ Poly(glycerol) does not increase blood viscosity,^87^ improves residence time in circulation,^87^ and does not produce ABC.^205^ Nonetheless, despite their valuable properties and regardless of the fact that these polymers provoke little, if any, immunogenic responses, by now none of them has been found superior to PEG with respect to enhancing the pharmacokinetic performance of LNPs.^98^ The advantages and limitations of the PEG alternatives used in drug delivery and bioconjugation have been carefully examined and discussed in detail.^28^

**Figure 7:**
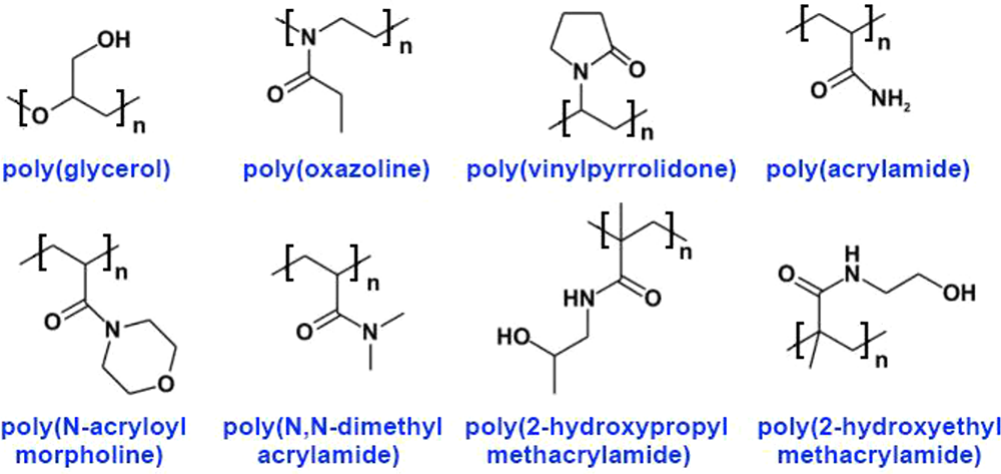
Structures of some polymers considered and tested as PEG alternatives.

The structures of some of the most studied PEG alternative polymers for use in pharmaceutical formulations are shown in Figure 7.

## Marketed Products Comprising PEG–Lipid LNPs and Products in Clinical Trials

PEG–lipids have been extensively used in pharmaceutical LNP formulations.^1,206^ Shortly after the beginning of the PEGylated LNPs exploration, adverse reactions such as hypersensitivity have been reported for PEGylated drug products. The risk of hypersensitivity reactions to liposomal doxorubicin formulations Doxil, Caelyx, and ThermoDox represents a major warning upon the drug prescriptions and is widely recognized for special attention.^207^ CARPA has been reported as a hypersensitivity reaction for liposomal drugs and vaccines (Table 6), for example, liposomal doxorubicin – Doxil (ALZA Corporation), Caelyx (Janssen Pharmaceutica), and ThermoDox (Celsion),^177,208,209^ as well as transthyretin-directed small inhibitory RNA-containing nanoparticle (Patisiran, Onpattro).^19^ Reported adverse reactions to Onivyde^210^ include anaphylactic reaction and hypersensitivity. Exemplary PEG–lipid structures used in marketed formulations are depicted in Figure 2.

**Table 6:** Clinically Approved LNP Formulations Comprising PEG–Lipid Conjugates and the Reported Adverse Effects^1,18,29,43,113,128,211−220^

trade name	approval	active ingredient	lipid composition^a^	indication for use	immuno-induced adverse effects
Doxil/Caelyx^221,222^	1995, 1996	Doxorubicin	HSPC:Chol:PEG2000–DSPE (56:39:5)	ovarian, breast cancer, Kaposi’s sarcoma	ABC, CARPA
ThermoDox^223^	2014	Doxorubicin	DPPC, MSPC, PEG2000–DSPE	hepatocellular carcinoma	CARPA
Onivyde^224−226^	2015	Irinotecan	DSPC:mPEG–2000:DSPE (3:2:0.015)	metastatic pancreatic adenocarcinoma	hypersensitivity, anaphylaxis
Onpattro (Patisiran)^227,228^	2018	RNAi, transthyretin-directed siRNA	DLin-MC3-DMA, PEG2000-C-DMG, DSPC, Chol	hATTR amyloidosis	CARPA
Lipoplatin^229^	2018	cisplatin	HSPC/DPPG/DSPE-mPEG2000	NSCLC, breast tumor, gastric tumor	N/A
BNT162b2 (Comirnaty; tozinameran)^40,230^	2021	mRNA	ALC-0315:ALC-0159:Chol:DSPC (46.3:1.6:42.7:9.4)	COVID-19 vaccine	anaphylaxis
mRNA-1273 (Spikevax)^38,41^	2021	mRNA	SM-102:PEG2000-DMG:Chol:DSPC (50:1.5:38.5:10)	COVID-19 vaccine	hypersensitivity, anaphylaxis

a Abbreviations: Chol, cholesterol; PC, phosphatidylcholine; PE, phosphatidylethanolamine; DPPC, dipalmitoyl PC; PG, phosphatidylglycerol; DSPC, distearoyl PC; DSPE, distearoyl PE; HSPC, hydrogenated soy PC; MSPC, monostearoyl PC; DPPG, dipalmitoyl PG; ALC-0315, (4-hydroxybutyl) azanediyl)bis (hexane-6,1-diyl) bis (2- hexyldecanoate); ALC-0159, 2- [(polyethylene glycol)- 2000]-N,N-ditetradecylacetamide; SM-102*(heptadecan-9-yl8-((2-hydroxyethyl)(6-oxo-6-(undecyloxy) hexyl) amino)octanoate).

**Figure 8:**
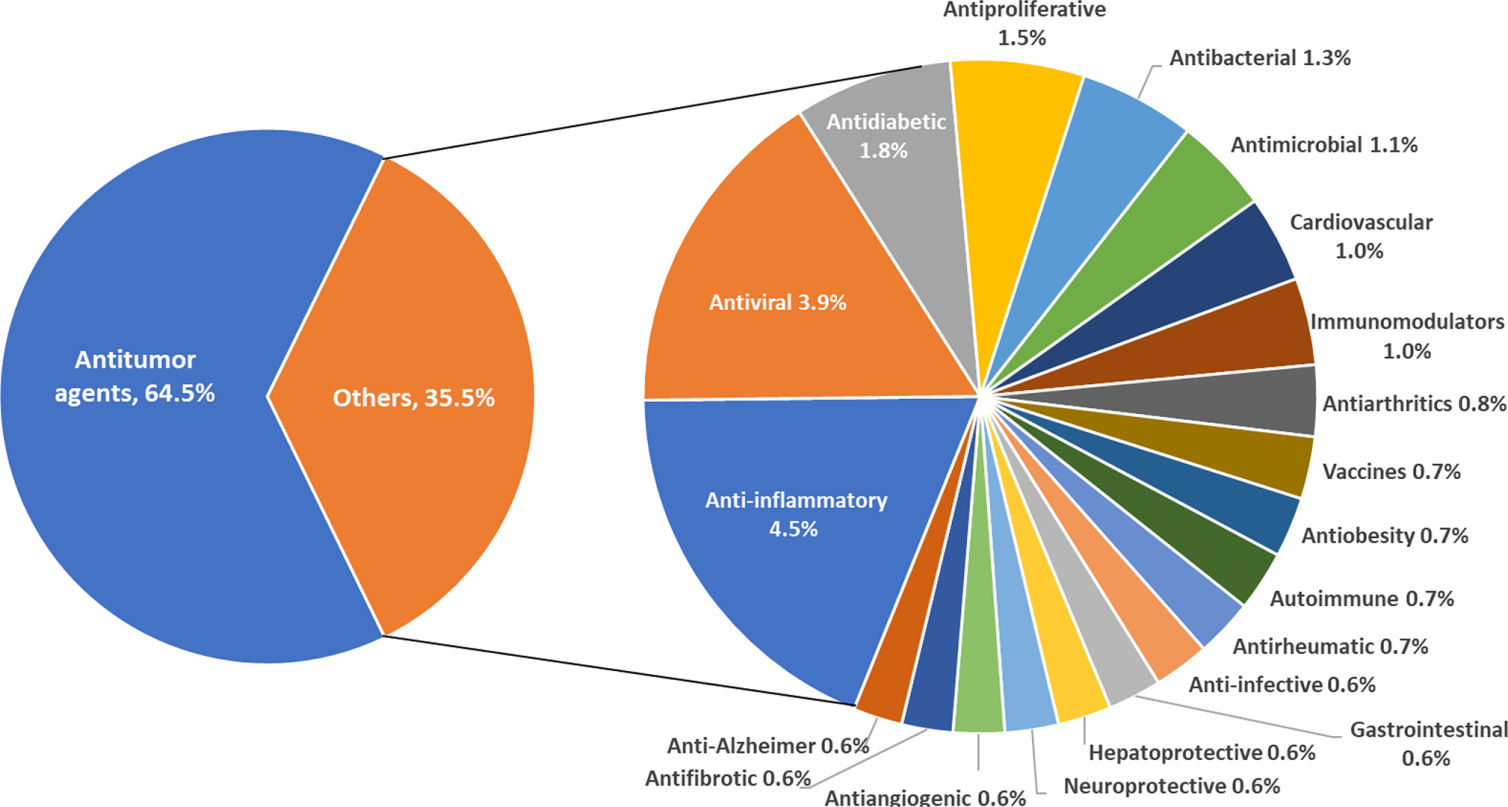
PEGylated LNP formulations explored as medications against various diseases and disorders according to the CAS Content Collection.

PEGylated LNP formulations are widely explored as medications against various diseases and disorders and are extensively represented in the CAS Content Collection. Nearly 2/3 of them are related to antitumor medicines (Figure 8). Anti-inflammatory and antiviral formulations are also highly represented. In many of these formulations, similar CARPA and hypersensitivity reactions concerns are being expressed.

Furthermore, currently over 200 clinical trials examining PEGylated lipid safety^231,232^ are listed on ClinicalTrials.gov. Over 60 of those studies are currently recruiting or in active status. Most of these studies are researching the safety of formulations comprising PEGylated liposomal doxorubicin in various solid tumors, along with both mRNA SARS-CoV-2 vaccines, Comirnaty and Spikevax. PEGylated vaccine safety and immunogenicity in individuals with varying degrees of immunosuppression is currently extensively researched in clinical trials. Exemplary clinical trials (phases I–IV) researching PEGylated lipid formulations safety in recruiting or active status are displayed in Table 7. Interventions include PEGylated liposomal formulations of doxorubicin, irinotecan, and retinoic acid for the treatment of various solid tumors, along with SARS-CoV-2 vaccines.

**Table 7:** Exemplary Recruiting/Active Status Clinical Trials (Phases I–IV) Researching PEGylated Lipid Formulation Safety

clinical trial identifier	interventions	conditions	status
NCT03483038	PEGylated Liposomal Irinotecan (Onivyde)/FOLFOX regimen	pancreatic cancer	recruiting
NCT05000216	Comirnaty/Spikevax	SARS-CoV-2	recruiting
NCT05029999	PEGylated Liposomal Doxorubicin (Doxil)	breast cancer	recruiting
NCT05077254	Comirnaty	SARS-CoV-2	recruiting
NCT05388487	PEGylated Liposomal All-Trans Retinoic Acid (HF1K16)	solid tumor	recruiting
NCT01210768	PEGylated Liposomal Doxorubicin/Cyclophosphamide	breast cancer	active, not recruiting
NCT02839707	PEGylated Liposomal Doxorubicin/Atezolizumab/Bevacizumab	ovarian, fallopian tube, and peritoneal cancer	active, not recruiting
NCT03088813	PEGylated Liposomal Irinotecan (Onivyde)/Topotecan	lung cancer	active, not recruiting
NCT04715438	Spikevax	SARS-CoV-2	active, not recruiting
NCT05618548	Comirnaty/Spikevax	SARS-CoV-2	active, not recruiting

Over 30 PEGylated lipid safety clinical trials report drug safety data.^231^ Examples of clinical trials with published results on PEGylated LNP safety are displayed in Table S2 and discussed in the text below.

Clinical trials (NCT01485874/NCT02163720)^233,234^ researching the treatment of ovarian cancer with liposomal doxorubicin formulations, Doxil and Caelyx, respectively, both revealed instances of hypersensitivity.^221,222^ Another clinical trial (NCT00826085)^235^ researching the safety of ThermoDox for the treatment of breast cancer also revealed hypersensitivity as a side effect.^223^ Small inhibitory RNA-based nanoparticle formulation, Patisiran, displayed hypersensitivity as well in clinical trials (NCT01961921/NCT03862807)^236,237^ for the treatment of transthyretin-mediated amyloidosis.^227,228^ In contrast, Onivyde, a nanoliposomal irinotecan formulation used to treat various solid tumors, failed to show immune-induced side effects in numerous clinical trials (NCT01770353, NCT03524508, and NCT03207724).^224−226^ 2B3-201, a glutathione-PEGylated liposome containing methylprednisolone for the treatment of multiple sclerosis, also did not show immune-induced side effects in a phase I trial with healthy subjects.^238^

SARS-CoV-2 vaccines, Comirnaty and Spikevax, both displayed immuno-induced adverse effects during clinical trials. Clinical trial NCT04368728 researching the safety, tolerability, immunogenicity, and efficacy of Comirnaty reported anaphylaxis as a side effect.^230^ Both anaphylaxis and hypersensitivity were reported results for clinical trial NCT04405076 researching the safety and immunogenicity of the Spikevax vaccine.^38^

## Outlook and Perspectives

LNPs have already been recognized as efficient carriers to deliver a large variety of pharmaceuticals. Appreciating the current advances in LNP technologies and recognizing the challenges that still need to be overcome will allow future improvement of the existing platforms along with addressing the current translational and regulatory limitations. Continued translational success will need regular communication and close collaboration between experts involved in all phases of pharmaceutical development of the LNP technologies, including pharmaceutical design and manufacturing, toxicological examination, as well as preclinical and clinical evaluation.

Immunosafety is a strategic issue in current research and development of nanomedicines such as LNPs. As stated in regulatory guidances (e.g., refs (239−241)), the prediction and prevention of adverse immune reactions represent unfulfilled medical needs. Indeed, the combination of complexity and individual variation of the immune system, increasingly complex nanomedicines, will give, as it can be expected, increasingly complex responses. The immune toxicology of nanomedicines is a largely unexplored research area at a broad intersection of nanotechnology, immunology, and pharmacology, which attracts increasing interest.

The development of an approach to reduce the immunogenicity of PEGylated nanocarriers without substantially jeopardizing their performance would be highly needed for the further development of this favorable drug delivery system. For this purpose, a thorough understanding of the correlations between PEG–lipids structural parameters and the immune-related adverse effects of the PEGylated LNPs and their biophysical and physiological background is an essential requirement. Such knowledge would enable composing the optimal pharmaceutical formulations diminishing and eliminating the undesirable immune reactions and enhancing the safety and efficiency of the PEGylated medicines.

## Supporting Information Available

Table S1, PEG–lipid representatives used in LNPs as found in the CAS Content Collection with structures and the respective number of documents; and Table S2, exemplary clinical trials researching PEGylated lipid formulation safety (PDF)
