# Supplementary material for: PEGylated Lipid Nanoparticle Formulations: Immunological Safety and Efficiency Perspective
Source: Bioconjug Chem. 2023 May 10;34(6):941–60. doi: 10.1021/acs.bioconjchem.3c00174 (PMC10190134; doi:10.1021/acs.bioconjchem.3c00174)
Supplement: Supplementary file 1 — bc3c00174_si_001.pdf [file bc3c00174_si_001.pdf]

Supporting Information

PEGylated Lipid Nanoparticle Formulations: Immunological Safety and Efficiency Perspective

Rumiana Tenchov, Janet M. Sasso, Qiongqiong Angela Zhou*

CAS, a division of the American Chemical Society
2540 Olentangy River Rd, Columbus, OH 43202, USA

*Corresponding author: qzhou@cas.org

Contents:

Table S1. PEG-lipid representatives used in LNPs as found in CAS Content Collection with structures and the respective number of documents

Table S2. Exemplary clinical trials researching PEGylated lipid formulations safety

Table S1. PEG-lipid representatives used in LNPs as found in CAS Content Collection with structures and the respective number of documents

PEG-Lipid	Common name	Chemical structure	CAS Registry Number	All documents #	Patents #
**PEG-phosphatidylethanolamines (PEG-PE)**					
Dilauroyl (12:0/12:0) PE-PEG	DLPE-PEG	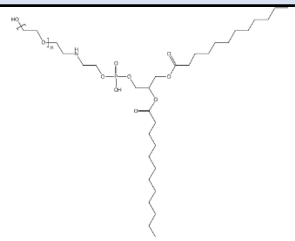	2055341-27-8	4	4

Dimyristoyl (14:0/14:0) PE-PEG	DMPE-PEG	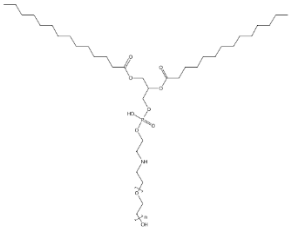	211567-66-7; 211733-74-3	107	53
Dipalmitoyl (16:0/16:0) PE-PEG	DPPE-PEG	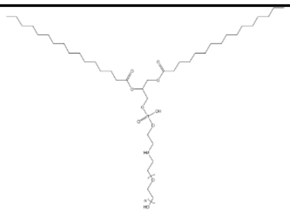	145035-97-8; 170931-03-0	267	142
Distearoyl (18:0/18:0) PE-PEG	DSPE-PEG	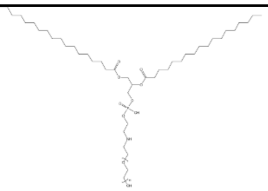	145035-96-7; 170931-04-1	1841	799
Oleoyl-Palmitoyl (18:1c9/16:0) PE-PEG	OPPE-PEG	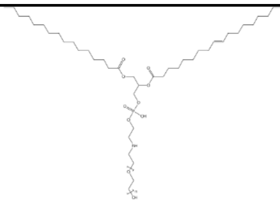	170127-34-1	6	5
Dioleoyl (18:1c9/18:1c9) PE-PEG	DOPE-PEG	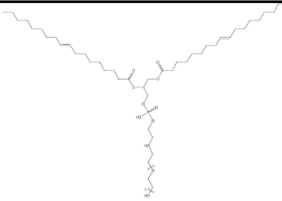	145035-95-6; 262601-19-4	116	60
Dilinoleoyl (18:2c9,12/18:2c9,12) PE-PEG	DLinPE-PEG	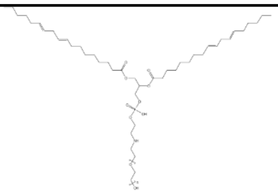	736998-47-3	4	4
**mPEG-phosphatidylethanolamines (mPEG-PE)**					
Dimyristoyl (14:0/14:0) PE-mPEG	DMPE-mPEG	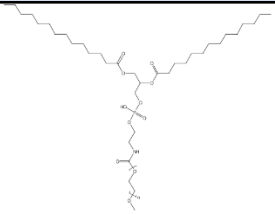	474922-82-2; 261764-82-3	101	65

Dipalmitoyl (16:0/16:0) PE-mPEG	DPPE-mPEG	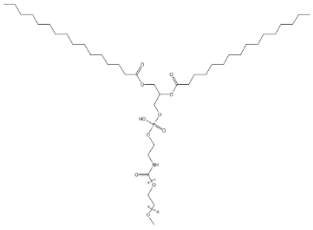	205494-72-0	91	50
Distearoyl (18:0/18:0) PE-mPEG	DSPE-mPEG	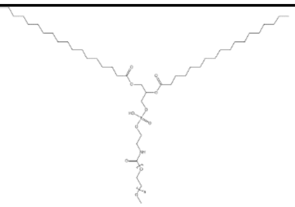	156543-00-9; 247925-28-6; 474922-77-5; 459428-35-4	2015	614
Dioleoyl (18:1c9/18:1c9) PE-mPEG	DOPE-mPEG	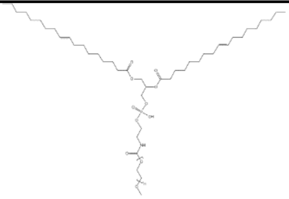	226940-29-0	53	26
**mPEG-glycerides**					
Dimyristoyl (14:0/14:0) glycerol-mPEG	DMG-PEG	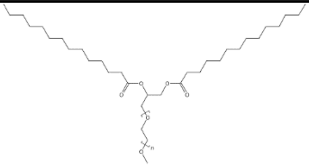	160743-62-4; 1397695-86-1	556	429
Dipalmioyl (16:0/16:0) glycerol-mPEG	PDG-PEG	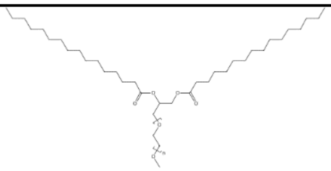	162409-28-1	31	25
Distearoyl (18:0/18:0) glycerol-mPEG	DSG-PEG; Sunbright DSG 2H	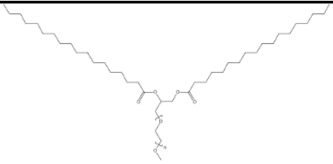	308805-39-2; 850628-36-3	76	50
Dioleoyl (18:1c9/18:1c9) glycerol-mPEG	DOG-PEG	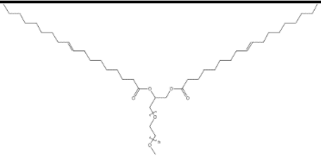	160743-61-3	6	5
**amino-mPEGs**					
Dilauroyl (12:0/12:0) amino-PEG		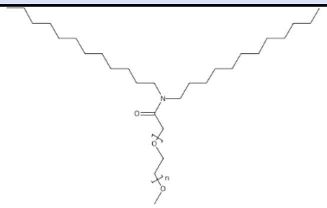	1849616-44-9	2	2

Lauroyl-Myristoyl (12:0/14:0) amino-PEG		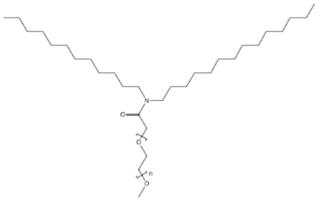	1849616-45-0	1	1
Dimyristoyl (14:0/14:0) amino-PEG	ALC-0159	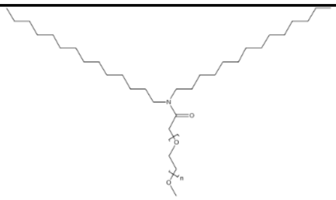	1849616-42-7	27	26
Dipalmitoyl (16:0/16:0) amino-PEG		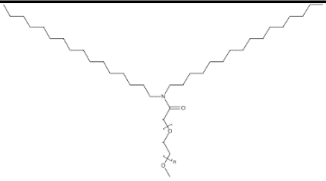	1849616-43-8	2	2
Distearoyl (18:0/18:0) amino-PEG		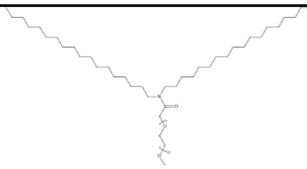	741737-56-4	3	2
**Chol-PEG**					
Cholesterol-PEG	PEG-cholesterol	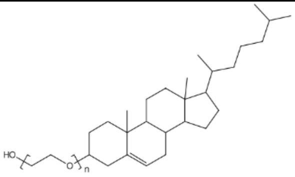	27321-96-6	626	460
Cholesterol-mPEG	mPEG-cholesterol	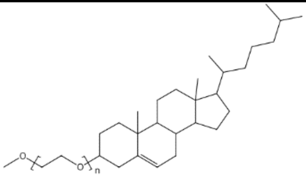	99559-58-7	27	14
Cholesterol-PEG-amine	Chol-PEG-NH_2	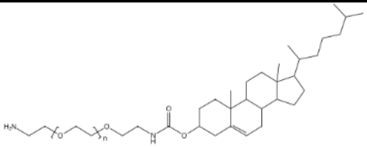	444045-24-3	11	3
PEG-cholesteryl carbonate	PEG-CHMC	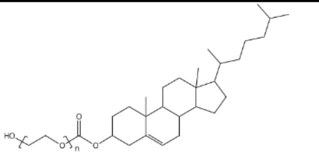	146185-41-3	5	1
**mPEG-ceramides**					

N-octanoyl-sphingosine-mPEG	C8 PEG Ceramide	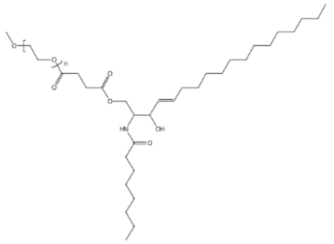	212116-76-2	31	20
N-palmitoyl-sphingosine-mPEG	C16 PEG Ceramide	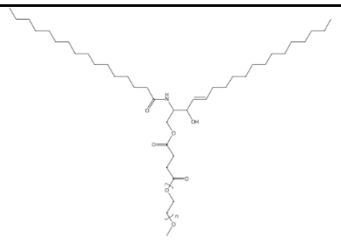	212116-78-4	85	38
**Functionalized PEG-lipids**					
Dioleloyl-PE-N-[carbonyl-amino-PEG]	DOPE-PEG Amine	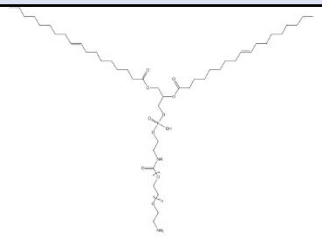	2342575-85-1	2	1
Dioleoyl-PE-N-[acetyl-PEG-O-acetic acid]	DOPE-PEG Carboxylic acid	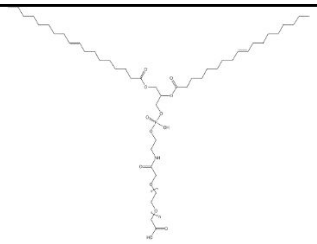	2342575-87-3	N/A	N/A
Bis-distearoyl-PE-N,N’-[diacetyl-PEG]	Bis-DSPE PEG	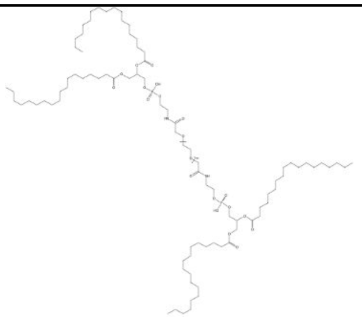	2260795-82-0	N/A	N/A
Distearoly-PE-N-[carbonyl-amino-PEG-N’-[4-(3-ethoxycyclobutane-1,2-dione)]]	DSPE-PEG-square	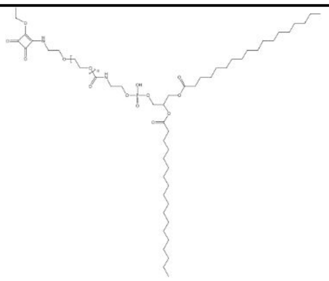	2315262-14-5	N/A	N/A

**Alternatives: Polysarcosine Lipids**					
N-tetradecyl polysarcosine		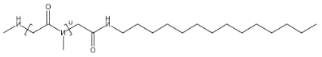	120468-71-5	2	1
N-Hexadecyl polysarcosine		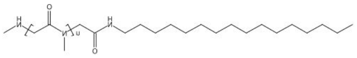	120468-76-0	2	1
N,N-ditetradecylamine-N-succinyl[methyl(polysarcosine)]	N-Tetamine-pSar45	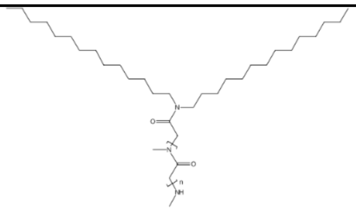	2847775-93-1	N/A	N/A

Table S2. Exemplary clinical trials researching PEGylated lipid formulations safety

Clinical Trial Identifier	Intervention	Active Ingredient	Indication for use	Immuno-induced adverse effects
NCT00826085	ThermoDox	Doxorubicin	Breast Cancer	Hypersensitivity
NCT01485874	Doxil	Doxorubicin	Ovarian cancer	Hypersensitivity
NCT01961921, NCT03862807	Patisiran	RNAi, transthyretin-directed siRNA	Transthyretin Mediated Amyloidosis	Hypersensitivity
NCT01770353, NCT03524508, NCT03207724	Onivyde	Irinotecan	Breast Cancer, Biliary Tract Cancer, Pancreatic Cancer	n/a
NCT02163720	Caelyx	Doxorubicin	Ovarian Cancer	Hypersensitivity
NCT04368728	Comirnaty	mRNA	SARS-CoV-2 Infection	Anaphylaxis
NCT04405076	Spikevax	mRNA	SARS-CoV-2 Infection	Anaphylaxis, Hypersensitivity
n/a	2B3–201	Methylprednisolone	Multiple Sclerosis	n/a
